# High Connectivity of the Crocodile Shark between the Atlantic and Southwest Indian Oceans: Highlights for Conservation

**DOI:** 10.1371/journal.pone.0117549

**Published:** 2015-02-17

**Authors:** Bruno Lopes da Silva Ferrette, Fernando Fernandes Mendonça, Rui Coelho, Paulo Guilherme Vasconcelos de Oliveira, Fábio Hissa Vieira Hazin, Evgeny V. Romanov, Claudio Oliveira, Miguel Neves Santos, Fausto Foresti

**Affiliations:** 1 Laboratório de Biologia e Genética de Peixes, Instituto de Biociências de Botucatu, Universidade Estadual Paulista, UNESP, Brasil; 2 Departamento de Ciências do Mar, Instituto do Mar, Universidade Federal de São Paulo, UNIFESP, Santos, Brasil; 3 Instituto Português do Mar e da Atmosfera, IPMA, IP, Olhão, Portugal; 4 Centro de Ciências do Mar, CCMAR, Universidade Algarve, Faro, Portugal; 5 Departamento de Pesca e Aquicultura, Universidade Federal Rural de Pernambuco, UFRPE, Pernambuco, Recife, Brasil; 6 Centre Technique d'Appui à la Pêche RéUNionnaise (CAP RUN), Association Réunionnaise de Développement de l'Aquaculture (ARDA), Le Port, Île de la Réunion, France; Ecole normale superieure de Lyon, FRANCE

## Abstract

Among the various shark species that are captured as bycatch in commercial fishing operations, the group of pelagic sharks is still one of the least studied and known. Within those, the crocodile shark, *Pseudocarcharias kamoharai*, a small-sized lamnid shark, is occasionally caught by longline vessels in certain regions of the tropical oceans worldwide. However, the population dynamics of this species, as well as the impact of fishing mortality on its stocks, are still unknown, with the crocodile shark currently one of the least studied of all pelagic sharks. Given this, the present study aimed to assess the population structure of *P. kamoharai* in several regions of the Atlantic and Indian Oceans using genetic molecular markers. The nucleotide composition of the mitochondrial DNA control region of 255 individuals was analyzed, and 31 haplotypes were found, with an estimated diversity Hd = 0.627, and a nucleotide diversity π = 0.00167. An analysis of molecular variance (AMOVA) revealed a fixation index *Φ*_ST_ = -0.01118, representing an absence of population structure among the sampled regions of the Atlantic Ocean, and between the Atlantic and Indian Oceans. These results show a high degree of gene flow between the studied areas, with a single genetic stock and reduced population variability. In panmictic populations, conservation efforts can be concentrated in more restricted areas, being these representative of the total biodiversity of the species. When necessary, this strategy could be applied to the genetic maintenance of *P. kamoharai*.

## Introduction

Among the various shark species commonly caught as bycatch in commercial fishing operations, the pelagic sharks are one of the groups in more need of attention, management and conservation actions [1]. In recent years, there has been a general increase in the commercial interest of those species, mainly due to the increased value of their meat and the value of their fins. However, and with a few exceptions (e.g. blue shark, *Prionace glauca*), this group of the pelagic sharks is still one of the least known and studied.

Currently, there is still a lack of definitive information about the current conservation status of most species [2, 3]; however, there is a general consensus that many populations are declining, with overfishing being one of the possible major causes [4]. Given that most species of sharks have low recruitment, slow growth rates, late sexual maturity and usually low fecundity, their populations tend to shown very low resilience against pressures such as fishing mortality, which can result in population declines and promote the reduction of the genetic variability levels of the populations.

The crocodile shark, *Pseudocarcharias kamoharai*, is a small pelagic shark species that inhabits from the sea surface to depths of at least 590 m in tropical and subtropical waters of the Atlantic, Indian and Pacific Oceans. It is usually found in the open sea, although it can sometimes occur in coastal waters [5], and in spite of its worldwide distribution, *P. kamoharai* has been poorly studied and not much is known about its biology [6]. The crocodile shark is occasionally caught as by-catch in industrial pelagic longline fleets operating in offshore waters, which are usually targeting large bony fishes such as tunas (Scombridae) and swordfish (Xiphiidae) [7]. Due to its very low or lack of commercial value, *P. kamoharai* specimens are usually discarded at the time of fishing gear retrieval, and are not retained for commercialization [8, 9]. As a result, the captures of *P. kamoharai* in commercial fisheries are rarely reported, and as such the available data to establish the potential impacts of the fisheries on those populations are also very limited.

In a recent study with an overview of pelagic sharks at-haulback mortality in longline fisheries in the Atlantic Ocean, [9] reported that the crocodile shark was the second most captured species, representing around 5% of the total elasmobranch catch, after the blue shark (*Prionace glauca*) that accounted for the large majority of the catches (84% of the elasmobranch catch). In that fishery, and considering the areas and seasons of operation of that particular fleet, the numbers of crocodile sharks caught were in the same order of magnitude of the shortfin mako shark (*Isurus oxyrinchus*), that represented around 4% of the total elasmobranch catch. This result highlights that in certain areas and seasons, the relative frequency of occurrence of *P. kamoharai* might be relatively high, but that those catches are probably unaccounted as the specimens are discarded.

The current lack of biological and fishery data on *P. kamoharai* opens many questions about the impact of the fisheries on these populations and ultimately about the conservation status of the species. Among the various sources of information needed to develop appropriate management and conservation plans, the study of the population genetics is an essential component, as it provides information regarding the stocks delimitation, biodiversity characterization, and identification of evolutionary units. Therefore, this study aimed to assess the genetic population structure of *P. kamoharai*, comparing specimens from the Atlantic and the southwestern Indian Oceans, in major areas of operation of pelagic longline fisheries.

## Materials and Methods

### Sample collection

**Fig 1:**
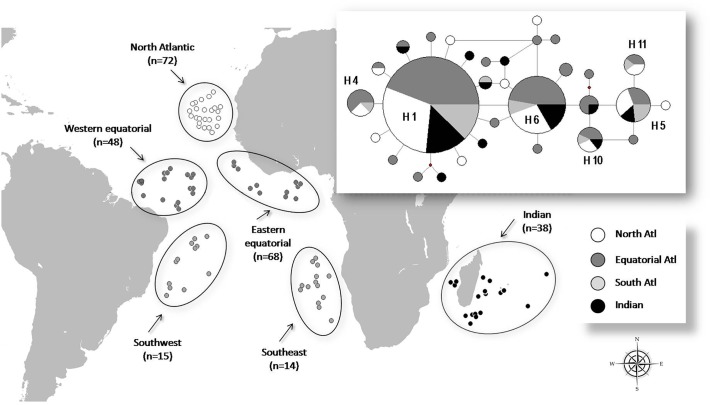
Geographic distribution of samples of *P. kamoharai*, with the network of haplotypes analyzed and compiled from the sequences of the mitochondrial DNA control region. Intermediate points in the network of haplotypes represent hypothetical haplotypes not sampled. All haplotypes differ by only a single mutation in the network, and the sizes of the circles are proportional to their frequencies.

The samples of the *P. kamoharai* were collected by fishery observers placed onboard commercial pelagic longline vessels from Portuguese, Brazilian and French fleets that operate in several regions of the Atlantic and southwest Indian Oceans (Fig. 1). For the French fleet based in Reunion Island, samples were also taken from crocodile sharks donated by vessels’ captains, and during a research cruise of the Institut Français de Recherche pour l'Exploitation de la Mer. The samples were collected opportunistically during the normal fishing operations of the vessels. Whenever a dead crocodile shark was captured in the longline, the fishery observer took a small muscle fragment (<1cm^3^) which was stored frozen in 96% ethanol. No specific permissions are required for collecting samples of crocodile sharks, a species currently not protected, from those locations/activities.

In total of 255 specimens were sampled, with 217 collected from different regions of the Atlantic Ocean, namely 48 from the western equatorial Atlantic, 72 from the northeast tropical Atlantic, 68 from the eastern equatorial Atlantic, 14 from the southeast Atlantic and 15 from the southwest Atlantic (Fig. 1). Another 38 samples were collected from the southwest Indian Ocean region. In each case, a small tissue sample (< 1 cm^2^) was collected from each specimen and preserved in 96% ethanol.

### DNA extraction, amplification and sequencing

Genomic DNA was extracted using the NucleoSpin Tissue XS Kit (Macherey & Nagel, Düren, Germany). Partial sequences of the mitochondrial control region were obtained according to the methodology used by [10]. Cycle sequencing was performed with the BigDye Terminator v3.1 Cycle Sequencing Kit (Applied Biosystems). Individual reactions were performed with approximately 30 ng template PCR product, 3.2 pmol primer, 1 μl terminator mix, and 5 μl Better Buffer (The Gel Co.) in a total volume of 15 μl. PCR sequencing profiles consisted of an initial denaturation step of 4min at 96°C, followed by 30 cycles of 30s at 96°C, 15s at 50°C, and 4 min at 60°C. Sequencing was carried out on an automated sequencer ABI 3130 Applied Biosystems, edited using BioEdit [11] and aligned using ClustalW [12]. The haplotype sequences were deposited in GenBank under accession numbers KF952742 to KF952772.

### Population analysis

The relative nucleotide composition, number of polymorphic sites, haplotype diversity (*h*), nucleotide diversity (π), and number of pairwise nucleotide differences among populations were calculated using ARLEQUIN 3.5.1.3 software [13]. To estimate the levels of genetic divergence among populations of *P. kamoharai*, the diversity measure *Φ*_ST_ was calculated using analysis of molecular variance (AMOVA) [14] under the parameters of the Tamura-Nei [15] nucleotide substitution model. *Φ*_ST_ estimates were non-parametrically tested (1000 bootstrapped replicates) using the ARLEQUIN 3.5.1.3 software, and adjusted for simultaneous pairwise comparisons using the sequential Bonferroni procedure [16]. A minimum-spanning haplotype network was estimated using the Network 4.611 program [17]. Evidence of population expansion was tested using Fu’s *F* test [18] and Tajima’s *D* [19] in ARLEQUIN 3.5.1.3. The mismatch analysis in ARLEQUIN 3.5.1.3 included a raggedness index to determine goodness-of-fit to a unimodal distribution [20].

## Results

The sequencing of the mitochondrial DNA control region of the 255 specimens of *P. kamoharai* resulted in 758 nucleotides that could be analyzed with 26 polymorphic sites, yielding 31 haplotypes (Table 1). The nucleotide frequencies were observed as Adenine = 31.94%, Thymine = 34.24%, Cytosine = 20.50% and Guanine = 13.31%. The total haplotype (*h*) and nucleotide (*π*) diversity were *h* = 0.627 and π = 0.00167, respectively, with the greatest diversities found in the eastern equatorial Atlantic Ocean (*h* = 0.69535, π = 0.00203), and in the southwest Indian Ocean (*h* = 0.64865, π = 0.00162). Haplotypes 1 and 6, shared by 154 and 36 specimens respectively, were the most common and were found on all the sampled regions, representing 74.5% of crocodile sharks analyzed (Table 2).

**Table 1:** Polymorphic nucleotide positions for *P. kamoharai* haplotypes.

	Nucleotide positions																									
Hap	1	2	2	2	2	2	2	2	2	2	2	3	3	3	3	3	3	3	3	3	4	4	4	4	4	4
	5	3	3	4	4	5	6	6	8	8	8	0	0	0	1	2	2	4	5	8	1	1	2	6	7	9
	5	6	8	0	4	4	5	6	0	7	9	0	6	7	6	3	7	4	8	8	6	8	4	5	3	8
**01**	T	G	T	T	A	A	A	A	A	C	T	C	C	C	G	C	T	T	G	C	A	T	C	G	G	T
**02**	.	A	.	.	.	.	.	.	.	.	.	.	.	T	.	.	.	A	.	.	.	.	.	.	.	C
**03**	.	.	.	.	.	.	.	.	.	.	.	.	.	.	.	.	.	.	.	.	.	.	T	.	.	.
**04**	.	.	.	.	.	.	.	.	.	.	C	.	.	.	.	.	.	.	.	.	.	.	.	.	.	.
**05**	.	.	.	.	.	.	.	.	.	.	C	.	.	.	.	.	.	.	.	.	.	.	.	.	.	C
**06**	.	.	.	.	.	.	.	.	.	.	.	.	.	.	.	.	.	.	.	.	.	.	.	.	.	C
**07**	.	.	.	.	.	.	.	.	.	.	.	.	.	.	.	.	.	.	.	.	.	.	.	.	.	A
**08**	.	.	.	.	.	.	.	.	.	.	.	.	.	.	.	.	.	A	.	.	.	.	.	.	.	C
**09**	.	.	.	.	.	.	.	.	.	.	.	A	.	.	.	.	.	.	.	.	.	.	.	.	.	C
**10**	.	A	.	.	G	.	.	.	.	.	.	.	.	.	.	.	.	.	.	.	.	.	.	.	.	C
**11**	.	A	C	.	.	.	.	.	.	.	C	.	.	.	.	.	.	.	.	.	.	.	.	.	.	C
**12**	.	.	.	.	.	.	.	.	T	.	.	.	.	.	.	.	.	.	.	.	.	.	.	.	.	.
**13**	.	.	.	C	.	.	.	.	.	.	.	.	.	.	.	.	.	.	.	.	.	.	.	.	.	C
**14**	.	.	.	.	.	.	.	.	.	A	.	.	.	.	.	.	.	.	.	.	.	.	.	.	.	.
**15**	.	.	.	.	.	.	G	.	.	.	.	.	.	.	.	.	.	A	.	.	.	.	.	.	.	.
**16**	.	A	.	.	.	T	.	.	.	.	C	.	.	.	.	.	.	.	.	.	.	.	.	.	.	C
**17**	.	.	.	.	.	.	.	.	.	.	.	.	.	.	.	.	.	A	.	.	.	.	.	.	.	C
**18**	.	.	.	.	.	.	.	.	.	.	.	.	.	.	.	T	.	.	.	.	.	.	.	.	.	.
**19**	.	.	.	C	G	.	.	.	.	.	.	.	.	.	.	.	.	A	.	.	.	.	.	.	.	C
**20**	.	A	.	.	.	.	.	.	.	.	.	.	.	.	.	.	.	.	.	.	.	.	.	.	.	C
**21**	.	.	.	.	.	.	.	.	.	.	.	.	T	.	.	.	.	.	.	.	.	.	.	.	.	C
**22**	.	.	.	.	.	.	.	.	.	.	.	.	.	T	.	.	.	.	.	.	.	.	.	.	.	.
**23**	.	.	.	.	.	.	.	.	.	.	.	.	.	.	A	.	.	A	.	.	.	.	.	.	.	C
**24**	.	.	.	.	.	.	.	.	.	.	.	.	.	.	.	.	.	.	A	T	T	G	.	A	A	.
**25**	.	A	.	.	G	.	.	.	.	.	C	.	.	.	.	.	.	.	.	.	.	.	.	.	.	C
**26**	.	A	C	.	.	.	.	.	.	.	.	.	A	.	.	.	.	.	.	.	.	.	.	.	.	.
**27**	.	.	.	C	.	.	.	.	.	.	.	.	.	.	.	.	.	.	.	.	.	.	.	.	.	.
**28**	.	.	.	C	.	.	.	.	.	.	.	.	.	.	.	.	.	A	.	.	.	.	.	.	.	C
**29**	C	.	.	.	.	.	.	.	.	.	.	.	.	.	.	.	.	.	.	.	.	.	.	.	.	.
**30**	.	.	.	.	.	.	.	T	.	.	.	.	.	.	.	.	.	.	.	.	.	.	.	.	.	.
**31**	.	A	C	.	.	.	.	.	.	.	.	.	.	.	.	.	C	.	.	.	.	.	.	.	.	.

**Table 2:** Geographical distribution of haplotypes of *P. kamoharai*, in number of specimens per region.

**Hap**	**Western equatorial Atlantic (48)**	**Eastern equatorial Atlantic (68)**	**Southeast Atlantic (14)**	**Southwest Atlantic (15)**	**Northeast tropical Atlantic (72)**	**Indian Ocean (38)**	**Total (255)**
01	33	35	10	9	45	22	154
02	1	.	.	.	.	.	1
03	1	.	.	.	.	.	1
04	3	2	.	1	2	.	8
05	1	3	2	1	4	2	13
06	3	14	1	2	10	6	36
07	1	.	.	.	.	.	1
08	1	.	.	.	.	.	1
09	1	1	.	.	.	.	2
10	1	2	.	1	2	1	7
11	1	1	.	1	2	.	5
12	1	.	.	.	1	.	2
13	.	.	.	.	1	.	1
14	.	.	.	.	1	.	1
15	.	.	.	.	1	.	1
16	.	.	.	.	1	.	1
17	.	.	.	.	1	.	1
18	.	.	.	.	1	.	1
19	.	1	.	.	.	.	1
20	.	3	.	.	.	1	4
21	.	1	.	.	.	.	1
22	.	1	.	.	.	1	2
23	.	1	.	.	.	.	1
24	.	1	.	.	.	.	1
25	.	1	.	.	.	.	1
26	.	1	.	.	.	.	1
27	.	.	1	.	.	1	2
28	.	.	.	.	.	1	1
29	.	.	.	.	.	1	1
30	.	.	.	.	.	1	1
**31**	.	.	.	.	.	1	1

Considering possible differences between the various regional groups in the Atlantic, the AMOVA results showed a lack of structure and non-significant differences. This non-significance in terms of genetic differentiation was observed when testing several hypothesis, including a population structuring between the northern and southern hemispheres (*Φ*_ST_ = 0.00125, p = 0.6121) and between the eastern and western Atlantic Ocean (*Φ*_ST_ = -0.0016, p = 0.5918). Several tests also were made at a finer scale within the five regional groups of the Atlantic Ocean. Likewise, the same analysis to test the hypothesis of structuring between the Atlantic and Indian Oceans also resulted in an index revealing the absence of population genetic differentiation (*Φ*_ST_ = -0.01118, p = 0.9955). When the indices of structuring were compared between pairs of regions, on all cases, there was not any evidence of population differentiation, even when comparing pairs between the Atlantic and Indian Oceans (Table 3).

**Table 3:** Differentiation (*F*_ST_) between pairs of sampled regions in the Atlantic and Indian Oceans.

	**Northeastern Atlantic**	**Western equatorial Atlantic**	**Eastern equatorial Atlantic**	**Southwest Atlantic**	**Southeast Atlantic**	**Southwest Indian Ocean**
Northeastern Atlantic	-	0.5495	0.3063	0.9909	0.6396	0.9189
Western equatorial Atlantic	-0.0035	-	0.0991	0.8828	0.7207	0.2973
Eastern equatorial Atlantic	0.0028	0.0276	-	0.8828	0.2432	0.7837
Southwest Atlantic	-0.0359	-0.0248	-0.0251	-	0.6666	0.9909
Southeast Atlantic	-0.0179	-0.0194	0.0158	-0.0343	-	0.5855
Southwest Indian Ocean	-0.0118	0.0054	-0.0110	-0.0348	-0.0155	-

Pairwise *F*_ST_ below diagonal and p-values above the diagonal.

**Fig 2:**
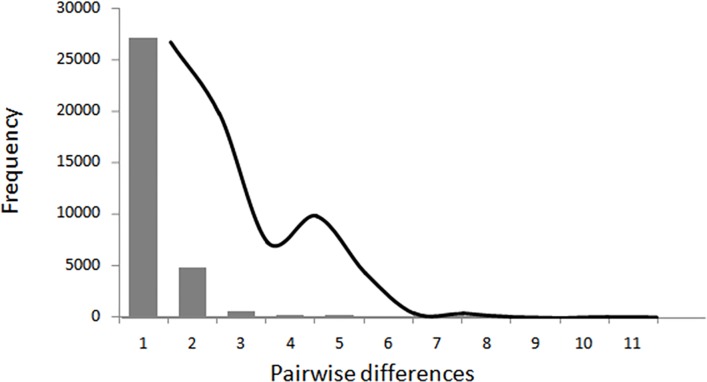
Pairwise mismatch distributions for overall sampled regions in the Atlantic and Indian Oceans. The observed values are represented in the in bars and the expected values in the line.

The neutrality tests, specifically the Tajima *D* and Fu *F*_S_, resulted in negative indices (overall Tajima's *D* = -1.911, overall Fu's *F*s = -29.226) with significant deviations from the null hypothesis of neutrality, suggesting the occurrence of an event of population expansion. In the evaluations for each sample group, the observed Tajima *D* ratios were close to zero on all groups, while for the Fu’s *F*_S_ tests such indices varied between -1.74 and -8.33 (Table 4). The Mismatch Distribution’s curve representing the historical demographics was close to zero with a slight bimodal configuration, suggesting a population bottleneck event with subsequent expansion. The population expansion event is also supported by the sum of the squared deviations (0.284, P>0.0001) and Harpending's index raggedness index (0.0548, P>0.001) (Fig. 2).

**Table 4:** *Pseudocarcharias kamoharai* population statistics—n, number of individuals; N, number of haplotypes; *h*, haplotype diversity; π, nucleotide diversity; *D*, value of Tajima D test; and F_S_, value of Fu test.

	**n**	**N**	***h***	**π**	**D**	**F_S_**
North Atlantic	72	12	0.59155	0.00157	-1.4429*	-7.25357
Western equatorial Atlantic	48	10	0.52660	0.00135	-1.57283	-8.33523
Eastern equatorial Atlantic	68	17	0.69535	0.00203	-1.69054	-8.07736
Southeast Atlantic	14	4	0.49451	0.00136	-0.5888*	-0.3499*
Southwest Atlantic	15	5	0.64762	0.00188	-0.2394*	-1.7462*
Indian Ocean	38	11	0.64865	0.00162	-1.62446	-6.32263
**Total**	**255**	**31**	**0.62712**	**0.00167**	**-1.91188**	**-29.2265**

* denotes statistical tests with p-values <0.05).

The network of haplotypes also suggests the absence of population structure with four haplotypes (H1, H5, H6 and H10) shared by the four major areas delimited as the North Atlantic, Equatorial Atlantic, South Atlantic and Indian Ocean. In the two major haplotypes with higher relative frequency (H1, H6), there is a derivation for a number of other rare haplotypes with only one mutation difference between the major haplotype and the derivatives, featuring a star network (Fig. 1).

## Discussion

### Population genetics

In recent years, with the use of molecular tools, there has been an increase in the availability of genetics data for many species, and information such as variability, population structure, gene flow and historical events have became widely considered in the conservation of threatened species and populations, and in the development of more efficient conservation plans.

In the present study the *P. kamoharai* indexes of nucleotide diversity were similar to reported for other pelagic shark with a single population as *Cetorhinus maximus* [21] and *Rhincodon typus* [22, 23]. When compared the indexes to other pelagic and coastal sharks many populations have haplotype and nucleotide diversities similar to or lower than that found in crocodile shark [24, 25, 26, 27, 28]. With the analysis of molecular variance, possible signals of structured populations in the Atlantic were not detected, even when considering various simulations in terms of hypothetical scenarios of differentiation. Thus, it can be assumed that the species *P. kamoharai* constitutes a single genetic stock with high gene flow throughout its distribution range in the Atlantic Ocean. However, there are some features that should be highlighted. While it is clear that there is a sharing of haplotypes by the various sampled regions, a higher nucleotide and haplotype diversity seems to be associated with the eastern equatorial region, closer to the Gulf of Guinea. Considering that there is no population structure among the overall sampled region, the slightly higher diversity in the eastern equatorial sample may just be due to variation among finite samples drawn from a single population. When the samples from the Indian Ocean were included in the AMOVA analysis similar results were obtained, with an absence of genetic structure differentiation between the Indian and all other groups of the Atlantic. Although the sampled area in the Indian Ocean is only representative of a relatively small area in the southwest Indian Ocean, the pattern of a high gene flow observed in almost all areas of occurrence of the species in the Atlantic, as well as the gene flow between the two Oceans, seems to point to the possibility of a single genetic stock of *P. kamoharai* distributed between these two Oceans basins, configuring a single population.

The negative indices found for the Tajima’s *D* test can mean an excess of low frequency polymorphisms in relation to expectations, observed in the high number of haplotypes with single representative (20), indicating an event of expansion of population size, that probably occurred after a bottleneck event or purification selection. However, the occurrence of shared haplotypes among the Atlantic and Indian Oceans may indicate a recent or contemporary dispersal, suggesting that these sharks cross waters off the southern tip of the African Continent, and support the continuous gene flow between these two Oceans.

In the haplotype network a configuration related to the absence of population genetic structure with three haplotypes represented in all sampled groups was observed. Considering the distribution of the sampled groups in four major areas, North Atlantic, Equatorial Atlantic, South Atlantic and Indian Ocean, the presence of four shared haplotypes was observed. Other 6 haplotypes are shared by at least two major areas. Haplotype 1, with the highest relative frequency (60.4%) was represented by individuals on all sampled areas, including the Indian Ocean, being found in greater numbers in the North Atlantic (n = 45). However, the frequency of this haplotype was observed in their majority in the South Atlantic (71.4%). The haplotype 1 had many derived less frequent haplotypes, setting a star configuration that suggests a recent process of population expansion after a bottleneck event.

### Conservation

In the last years sharks have gained some relative importance in terms of commercial value, mainly due to the increasing value of their meat and fins. In the particular case of the crocodile shark, and even though this species has no commercial value and is therefore usually discarded, there is still fishing mortality taking place and impacting the populations, as part of the catch is captured and discarded already dead [9]. Therefore, this shark constitutes a particular species where no conservation and/or management initiatives are usually considered or implemented.

Genetic factors influence the risk of extinction mainly because endangered species usually have small and/or declining populations, and in such populations inbreeding and loss of genetic diversity will inevitably put the populations at risk, with evolutionary potential lost even before they may eventually become effectively extinct. These results for *P. kamoharai* suggest the occurrence of a single stock, without a strong differentiation in haplotype frequencies, forming one basic unit of management in the Atlantic and possibly, in the Indian Ocean. With an absence of population genetic structure, genetic diversity similar or higher than that found in many shark species, although still without consideration of conservation measures, attention with a continuing monitoring through time and an evaluation of the effects caused by fishing pressure are recommended.
